# Pimelic acid–urea (1/2)

**DOI:** 10.1107/S1600536811023439

**Published:** 2011-06-25

**Authors:** Wei Xu, Wen-xiang Huang, Hong-yan Chen

**Affiliations:** a Center of Applied Solid State Chemistry Research, Ningbo University, Ningbo, Zhejiang 315211, People’s Republic of China

## Abstract

The asymmetric unit, 2CH_4_N_2_O·C_7_H_12_O_4_, of the title cocrystal contains one urea mol­ecule and a half-mol­ecule of pimelic acid; the latter, together with a second urea mol­ecule, are completed by symmetry, with the central atom of the whole pimelic acid moiety placed on a twofold crystallographic axis. The crystal packing is stabilized by O—H⋯O and N—H⋯O hydrogen-bond, generating a chain along [10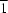]. Additionally, the chains are assembled into a three-dimensional framework *via* weak N—H⋯O inter­chain inter­actions.

## Related literature

For urea inclusion compounds, see: Videnova-Adrabińska (1996*a* ▶); Harris & Thomas (1990 ▶); Yeo *et al.* (1997 ▶). For urea-dicarb­oxy­lic acid co-crystal engineering with predesigned crystal building blocks, see: Videnova-Adrabińska (1996*b* ▶); Chadwick *et al.* (2009 ▶); Chang & Lin (2011 ▶).
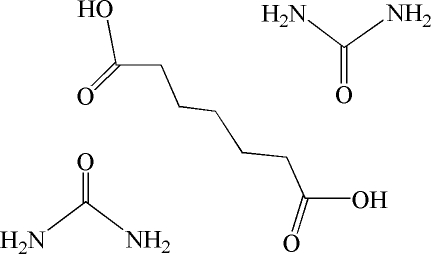


## Experimental

### 

#### Crystal data

2CH_4_N_2_O·C_7_H_12_O_4_
*M* *_r_* = 280.29
Monoclinic, 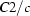

*a* = 15.103 (3) Å
*b* = 11.073 (2) Å
*c* = 9.1660 (18) Å
β = 112.72 (3)°
*V* = 1413.9 (6) Å^3^
*Z* = 4
Mo *K*α radiation
μ = 0.11 mm^−1^
*T* = 293 K
0.14 × 0.12 × 0.10 mm

#### Data collection

Rigaku R-AXIS RAPID diffractometer
Absorption correction: multi-scan (*ABSCOR*; Higashi, 1995 ▶) *T*_min_ = 0.989, *T*_max_ = 0.989
6742 measured reflections
1609 independent reflections
1084 reflections with *I* > 2σ(*I*)
*R*_int_ = 0.028

#### Refinement

*R*[*F* ^2^ > 2σ(*F* ^2^)] = 0.043
*wR*(*F* ^2^) = 0.135
*S* = 1.14
1609 reflections
87 parameters
H-atom parameters constrained
Δρ_max_ = 0.18 e Å^−3^
Δρ_min_ = −0.17 e Å^−3^

### 

Data collection: *RAPID-AUTO* (Rigaku, 1998 ▶); cell refinement: *RAPID-AUTO*; data reduction: *CrystalStructure* (Rigaku/MSC, 2004 ▶); program(s) used to solve structure: *SHELXS97* (Sheldrick, 2008 ▶); program(s) used to refine structure: *SHELXL97* (Sheldrick, 2008 ▶); molecular graphics: *ORTEPII* (Johnson, 1976 ▶); software used to prepare material for publication: *SHELXL97*.

## Supplementary Material

Crystal structure: contains datablock(s) global, I. DOI: 10.1107/S1600536811023439/lr2013sup1.cif

Structure factors: contains datablock(s) I. DOI: 10.1107/S1600536811023439/lr2013Isup2.hkl

Supplementary material file. DOI: 10.1107/S1600536811023439/lr2013Isup3.cml

Additional supplementary materials:  crystallographic information; 3D view; checkCIF report

## Figures and Tables

**Table 1:** Hydrogen-bond geometry (Å, °)

*D*—H⋯*A*	*D*—H	H⋯*A*	*D*⋯*A*	*D*—H⋯*A*
O1—H1⋯O3	0.88	1.72	2.584 (2)	168
N1—H1*A*⋯O2	0.86	2.24	3.038 (2)	154
N1—H1*B*⋯O2^i^	0.86	2.24	3.016 (2)	151
N2—H2*C*⋯O3^ii^	0.86	2.11	2.952 (2)	167
N2—H2*D*⋯O2^i^	0.86	2.49	3.211 (2)	142

Symmetry codes: (i) 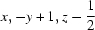; (ii) 
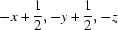
.

**Fig. 1.**
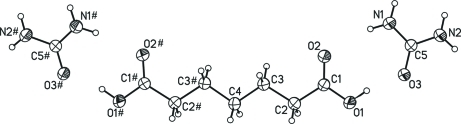
ORTEP view of the title co-crystal. The displacement ellipsoids are drawn at 35% probability level. (# = -x, y, -z + 3/2)

**Fig. 2.**
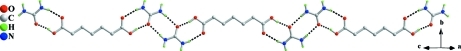
One-dimensional chain of the title co-crystal viewed along [101] direction.O—H···O and N—H···O hydrogen bonds are shown as dashed line.

**Fig. 3.**
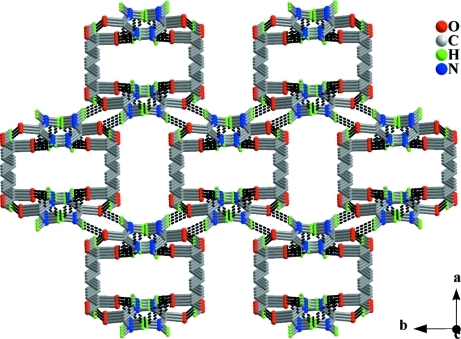
A view of the three-dimensional crystal structure of the title co-crystal viewed down the c axis.

## supplementary crystallographic information

### Comment

This crystal structure study is part of a broader program of urea-dicarboxylic acid co-crystal engineering with predesigned crystal building blocks (Videnova-Adrabińska, 1996**a*,b*; Chang & Lin, 2011). In these solids, the urea molecules form an extensively hydrogen-bonded host structure (Harris & Thomas,1990), containing linear, parallel tunnels with guest molecules packed densely along these tunnels (Yeo *et al.*, 1997). The phase diagram of a related urea- dicarboxylic acid co-crystal has also been reported (Chadwick *et al.* 2009). In this contribution, we present the crystal structure of the 2:1 urea/pimelic acid co-crystal.

The asymmetric unit of the title co-crystal, CH_4_N_2_O. 0.5(C_7_H_12_O_4_), contains one urea molecule and a half-molecule of pimelic acid, with the complete pimelic acid molecule and the additional urea unit generated *via* crystallographic rotation symmetry, with the central carbon atom of the whole pimelic acid molecule positioned on a twofold axis (Fig. 1).

Five different hydrogen-bond interactions (Table.1), organize the parent molecules in a well developed three-dimensional crystal structure. The carboxylic groups of the acid connect with the corresponding urea and inter-urea molecules through O1—H1··· O3, N1—H1A .. .O2 and N2—H2C ···O3 hydrogen bonds (Table 1), generating a one dimensional chain along [101] (Figure 2). Additional weak inter-chain N—H···O intermolecular interactions (Table 1) generated a three-dimensional network, which stabilizes the crystal packing (Figure 3).

.

### Experimental

Pimelic acid acid (0.0815 g, 0.5 mmol) and urea (0.0316 g, 0.05 mmol) were dissolved in 15 ml of water (pH = 3.23) under stirring. After slow evaporation of the solution for one week at 50°C, colorless block sized crystals were obtained.

### Refinement

H atoms bonded to C and N atoms were placed in their geometrically calculated position and refined using a riding model, with C-H distances 0.97 Å and N-H distances 0.86 Å and *U*_iso_(H) = 1.2 *U*_eq_(C,N). H atoms attached to O atoms were found in a difference Fourier synthesis and refined using a riding model, with the O—H distances fixed as initially found and with *U*_iso_(H) values set at 1.2 *U*_eq_(O).

### Crystal data

**Table:**

2CH_4_N_2_O·C_7_H_12_O_4_	*F*(000) = 600
*M**_r_* = 280.29	*D*_x_ = 1.317 Mg m^−^^3^
Monoclinic, *C*2/*c*	Mo *K*α radiation, λ = 0.71073 Å
Hall symbol: -C 2yc	Cell parameters from 6742 reflections
*a* = 15.103 (3) Å	θ = 3.7–27.4°
*b* = 11.073 (2) Å	µ = 0.11 mm^−^^1^
*c* = 9.1660 (18) Å	*T* = 293 K
β = 112.72 (3)°	Block, colorless
*V* = 1413.9 (6) Å^3^	0.14 × 0.12 × 0.10 mm
*Z* = 4	

### Data collection

**Table:**

Rigaku R-AXIS RAPID diffractometer	1609 independent reflections
Radiation source: fine-focus sealed tube	1084 reflections with *I* > 2σ(*I*)
graphite	*R*_int_ = 0.028
Detector resolution: 0 pixels mm^-1^	θ_max_ = 27.4°, θ_min_ = 3.7°
ω scans	*h* = −19→18
Absorption correction: multi-scan (*ABSCOR*; Higashi, 1995)	*k* = 0→14
*T*_min_ = 0.989, *T*_max_ = 0.989	*l* = 0→11
6742 measured reflections	

### Refinement

**Table:**

Refinement on *F*^2^	Primary atom site location: structure-invariant direct methods
Least-squares matrix: full	Secondary atom site location: difference Fourier map
*R*[*F*^2^ > 2σ(*F*^2^)] = 0.043	Hydrogen site location: inferred from neighbouring sites
*wR*(*F*^2^) = 0.135	H-atom parameters constrained
*S* = 1.14	*w* = 1/[σ^2^(*F*_o_^2^) + (0.0612*P*)^2^ + 0.4165*P*] where *P* = (*F*_o_^2^ + 2*F*_c_^2^)/3
1609 reflections	(Δ/σ)_max_ < 0.001
87 parameters	Δρ_max_ = 0.18 e Å^−^^3^
0 restraints	Δρ_min_ = −0.17 e Å^−^^3^

### Special details

**Table:**

Geometry. All esds (except the esd in the dihedral angle between two l.s. planes) are estimated using the full covariance matrix. The cell esds are taken into account individually in the estimation of esds in distances, angles and torsion angles; correlations between esds in cell parameters are only used when they are defined by crystal symmetry. An approximate (isotropic) treatment of cell esds is used for estimating esds involving l.s. planes.
Refinement. Refinement of *F*^2^ against ALL reflections. The weighted *R*-factor *wR* and goodness of fit *S* are based on *F*^2^, conventional *R*-factors *R* are based on *F*, with *F* set to zero for negative *F*^2^. The threshold expression of *F*^2^ > σ(*F*^2^) is used only for calculating *R*-factors(gt) *etc*. and is not relevant to the choice of reflections for refinement. *R*-factors based on *F*^2^ are statistically about twice as large as those based on *F*, and *R*- factors based on ALL data will be even larger.

### Fractional atomic coordinates and isotropic or equivalent isotropic displacement parameters (Å^2^)

**Table:**

	*x*	*y*	*z*	*U*_iso_*/*U*_eq_	Occ. (<1)
O1	0.13778 (11)	0.15952 (10)	0.34879 (17)	0.0693 (5)	
H1	0.1579	0.2051	0.2889	0.104*	
O2	0.11144 (11)	0.33586 (10)	0.43696 (16)	0.0690 (5)	
C1	0.10938 (12)	0.22682 (14)	0.4400 (2)	0.0449 (4)	
C2	0.07317 (13)	0.15469 (14)	0.5438 (2)	0.0502 (4)	
H2A	0.1236	0.1001	0.6073	0.060*	
H2B	0.0195	0.1058	0.4771	0.060*	
C3	0.04121 (12)	0.22885 (14)	0.6531 (2)	0.0445 (4)	
H3A	−0.0070	0.2863	0.5906	0.053*	
H3B	0.0956	0.2743	0.7244	0.053*	
C4	0.0000	0.1528 (2)	0.7500	0.0470 (6)	
H4A	−0.0501	0.1012	0.6793	0.056*	0.50
H4B	0.0501	0.1012	0.8207	0.056*	0.50
O3	0.19575 (9)	0.26507 (10)	0.15014 (14)	0.0545 (4)	
N1	0.13952 (13)	0.45279 (13)	0.1580 (2)	0.0679 (5)	
H1A	0.1134	0.4302	0.2218	0.082*	
H1B	0.1347	0.5267	0.1270	0.082*	
N2	0.22675 (12)	0.41306 (13)	0.00916 (19)	0.0613 (5)	
H2C	0.2583	0.3644	−0.0257	0.074*	
H2D	0.2205	0.4876	−0.0194	0.074*	
C5	0.18758 (12)	0.37311 (14)	0.10736 (19)	0.0449 (4)	

### Atomic displacement parameters (Å^2^)

**Table:**

	*U*^11^	*U*^22^	*U*^33^	*U*^12^	*U*^13^	*U*^23^
O1	0.1148 (12)	0.0399 (7)	0.0885 (10)	0.0013 (7)	0.0783 (10)	−0.0003 (6)
O2	0.1195 (12)	0.0373 (7)	0.0791 (10)	−0.0059 (7)	0.0702 (9)	−0.0022 (6)
C1	0.0563 (10)	0.0379 (8)	0.0491 (9)	−0.0027 (7)	0.0299 (8)	−0.0030 (7)
C2	0.0674 (11)	0.0394 (8)	0.0559 (10)	−0.0036 (7)	0.0370 (9)	−0.0001 (7)
C3	0.0529 (10)	0.0399 (8)	0.0483 (9)	−0.0007 (7)	0.0280 (8)	−0.0001 (7)
C4	0.0575 (14)	0.0411 (12)	0.0520 (13)	0.000	0.0319 (12)	0.000
O3	0.0794 (9)	0.0391 (6)	0.0644 (8)	0.0060 (6)	0.0493 (7)	0.0062 (5)
N1	0.1134 (14)	0.0391 (8)	0.0793 (11)	0.0103 (8)	0.0681 (11)	0.0039 (7)
N2	0.0924 (12)	0.0416 (8)	0.0735 (11)	0.0023 (7)	0.0579 (10)	0.0069 (7)
C5	0.0588 (10)	0.0376 (8)	0.0447 (9)	−0.0023 (7)	0.0270 (8)	−0.0024 (7)

### Geometric parameters (Å, °)

**Table:**

O1—C1	1.3099 (19)	C4—C3^i^	1.5208 (19)
O1—H1	0.8820	C4—H4A	0.9700
O2—C1	1.2085 (19)	C4—H4B	0.9700
C1—C2	1.498 (2)	O3—C5	1.2501 (18)
C2—C3	1.512 (2)	N1—C5	1.335 (2)
C2—H2A	0.9700	N1—H1A	0.8592
C2—H2B	0.9700	N1—H1B	0.8599
C3—C4	1.5208 (19)	N2—C5	1.329 (2)
C3—H3A	0.9700	N2—H2C	0.8592
C3—H3B	0.9700	N2—H2D	0.8600
			
C1—O1—H1	110.4	C3^i^—C4—C3	112.75 (18)
O2—C1—O1	122.40 (15)	C3^i^—C4—H4A	109.0
O2—C1—C2	124.51 (14)	C3—C4—H4A	109.0
O1—C1—C2	113.08 (13)	C3^i^—C4—H4B	109.0
C1—C2—C3	114.81 (13)	C3—C4—H4B	109.0
C1—C2—H2A	108.6	H4A—C4—H4B	107.8
C3—C2—H2A	108.6	C5—N1—H1A	119.9
C1—C2—H2B	108.6	C5—N1—H1B	120.1
C3—C2—H2B	108.6	H1A—N1—H1B	120.0
H2A—C2—H2B	107.5	C5—N2—H2C	120.0
C2—C3—C4	113.19 (13)	C5—N2—H2D	120.1
C2—C3—H3A	108.9	H2C—N2—H2D	120.0
C4—C3—H3A	108.9	O3—C5—N2	120.99 (15)
C2—C3—H3B	108.9	O3—C5—N1	121.64 (15)
C4—C3—H3B	108.9	N2—C5—N1	117.36 (15)
H3A—C3—H3B	107.8		
			
O2—C1—C2—C3	−2.1 (3)	C1—C2—C3—C4	176.91 (13)
O1—C1—C2—C3	179.01 (16)	C2—C3—C4—C3^i^	−174.14 (17)

Symmetry codes: (i) −*x*, *y*, −*z*+3/2.

### Hydrogen-bond geometry (Å, °)

**Table:**

*D*—H···*A*	*D*—H	H···*A*	*D*···*A*	*D*—H···*A*
O1—H1···O3	0.88	1.72	2.584 (2)	168.
N1—H1A···O2	0.86	2.24	3.038 (2)	154.
N1—H1B···O2^ii^	0.86	2.24	3.016 (2)	151.
N2—H2C···O3^iii^	0.86	2.11	2.952 (2)	167.
N2—H2D···O2^ii^	0.86	2.49	3.211 (2)	142.

Symmetry codes: (ii) *x*, −*y*+1, *z*−1/2; (iii) −*x*+1/2, −*y*+1/2, −*z*.
